# Interaction of the *Wolbachia* surface protein with a novel pro-viral protein from *Aedes aegypti*

**DOI:** 10.1128/mbio.01486-24

**Published:** 2024-11-22

**Authors:** Mazhar Hussain, Zhi Qi, Sassan Asgari

**Affiliations:** 1 Australian Infectious Disease Research Centre, School of Biological Sciences, The University of Queensland, Brisbane, Queensland, Australia; Stony Brook University, Stony Brook, New York, USA

**Keywords:** *Aedes aegypti*, mosquito, dengue virus, *Wolbachia*, serine threonine kinase

## Abstract

Dengue virus (DENV) and other flaviviruses are prevented from replicating in mosquitoes by *Wolbachia*. To date, several reports have appeared that highlight multiple molecular and cellular pathways involved in the blocking mechanism, which underlines the complicated nature of the mechanism. Here, we developed a hypothesis on whether *Wolbachia* proteins interact with pro-viral host proteins by using a unique approach to study the antiviral mechanism based on *Wolbachia*-host protein-protein interaction. We selected *Wolbachia* surface protein (WSP) for co-immunoprecipitation because of its abundance and possible secretion. We first confirmed WSP’s secretion in mosquito cells and found two host proteins, *Ae. aegypti* serine-threonine kinase (STK) and synaptic vesicle membrane (SVM) protein VAT-1, and one *Wolbachia* protein (*w*GroEL) interacting with WSP. We examined the role of *STK* and *SVM* genes in relation to DENV replication in *Ae. aegypti* mosquitoes and mosquito cell lines with and without *Wolbachia*. In DENV-infected Aag2 cells, the expression of *SVM* and *STK* was significantly increased. However, although these genes were induced in *Wolbachia*-infected Aag2 cells, they were downregulated after DENV infection. Silencing of *STK*, but not *SVM*, reduced DENV replication in Aag2 cells and mosquitoes. Conversely, RNA activation of *STK*, by utilizing promoter induction via short activating oligos, resulted in higher DENV replication in *Wolbachia*-infected and uninfected cell lines. Overall, our findings suggest that *STK* is a pro-viral gene, and *Wolbachia* WSP binds to STK, possibly making it less accessible for DENV replication.

**IMPORTANCE:**

*Wolbachia* is an endosymbiotic bacterium that blocks the replication of arboviruses in transinfected *Aedes aegypti* mosquitoes. In this study, we focused on identifying the potential interaction of *Wolbachia* proteins with the host pro-viral proteins. For this, we embarked on identifying the interacting proteins with a major *Wolbachia* protein, WSP, which is both structural and also secreted into the host cells. An *Ae. aegypti* STK was identified, which is induced in DENV and *Wolbachia*-infected cells. Silencing or induction of the gene led to reduced and increased DENV replication *in vitro*. Consistently, knocking down the gene in mosquitoes resulted in decreased virus replication. We hypothesize that WSP may sequester STK, which is pro-viral, contributing to *Wolbachia* virus blocking.

## INTRODUCTION

*Wolbachia* are maternally inherited endosymbiotic bacteria commonly found in various insect species, as well as nematodes, crustaceans, and arachnids (1, 2). They ensure efficient transmission by manipulating the host reproduction, primarily through cytoplasmic incompatibility, killing, or feminization of males (3). One remarkable attribute of a few strains of *Wolbachia* is their ability to inhibit the replication of medically important RNA viruses in mosquitoes (4, 5). *Wolbachia* mostly blocks the replication of positive-sense single-stranded RNA viruses, such as dengue virus (DENV), Zika, Chikungunya, and yellow fever viruses (YFVs). The primary vector of all these viruses is the mosquito, *Aedes aegypti*, which is not a natural carrier of *Wolbachia*. However, mosquitoes with lab-introduced *Wolbachia* strains exhibited low transmission of the abovementioned viruses (6–8).

Several publications have emerged on the potential mechanisms of *Wolbachia*-mediated antiviral resistance, reflecting the involvement of multiple mechanisms. Some of these mechanisms are interference in virus entry (9), viral RNA degradation (10, 11), competition for space (12) and resources (13), cholesterol sequestration to lipid droplets (14), and induction of host immune genes (15). All of these make an unfavorable milieu for virus replication. Since *Wolbachia* and flaviviruses share cellular resources for successful infection and propagation (16), they continuously compete to access resources. In this scenario, *Wolbachia* may effectively utilize their proteins in several ways to achieve this. For example, WalE1 co-localizes and bundles with actin and also binds to cellular Past1 protein to manipulate host endocytosis (17). Similarly, *Wolbachia* protein wBm0076 was found to co-localize with actin in nematodes and disrupt actin dynamics (18). In another report, *Wolbachia* was found to encode a conserved *Wolbachia* surface protein (wBm0152) in nematodes, which interacts with the host actin and tubulin to disrupt endosomal trafficking (19).

DENV infection causes severe diseases such as dengue haemorrhagic fever and dengue shock syndrome worldwide (20). DENV has an RNA genome of 10,700 nt that encodes a polyprotein on the endoplasmic reticulum membrane. The polyprotein is cleaved into three structural (capsid, prM, and envelope) and seven non-structural proteins (NS1, NS2A, NS2B, NS3, NS4A, NS4B, and NS5) (21). For successful transmission to a mammalian host, DENV requires efficient replication in their mosquito vector, which is mainly achieved through virus-host protein-protein interactions. The host proteins that assist virus replication are considered pro-viral proteins. A few pro-viral proteins have been identified in mosquitoes, such as SEC6 (22), loquaciou (23), and atlastin (24). RNAi-mediated silencing of all these genes significantly reduced DENV replication in mosquito cell lines.

In this study, we hypothesized if *Wolbachia* proteins interact with potentially pro-viral *Ae. aegypti* host proteins to contribute toward the virus-blocking mechanism. *Wolbachia* surface protein (WSP) is an abundant *Wolbachia* protein that not only partakes in the structure of *Wolbachia* but has also been shown to be secreted into the host cytoplasm in nematodes (19). By keeping those in mind, we chose WSP for co-immunoprecipitation (IP) assays to identify proteins that may interact with it through peptide sequencing. This led to the identification of two mosquito proteins and one *Wolbachia* protein. In the follow-up experiments, we investigated the role of the mosquito proteins in mosquito-*Wolbachia*-virus interactions using gene expression analysis, RNA interference, and RNA induction by utilizing promoter induction via short activating oligos. The outcomes shed light on the role of these proteins in DENV replication and how *Wolbachia* could manipulate them as part of its blocking mechanism.

## RESULTS

### *Wolbachia* WSP is secreted into the host cytoplasm in mosquito cells

**Fig 1:**
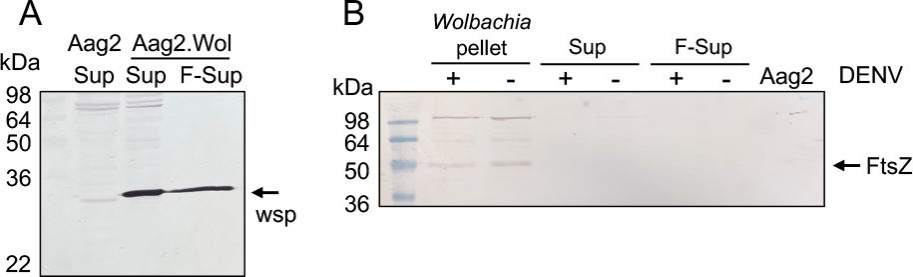
Secretion of *Wolbachia* WSP in mosquito Aag2.*w*MelPop cells. (**A**) Western blot analysis of the cytoplasmic fraction of Aag2 supernatant (Aag2 Sup) and *Wolbachia*-infected Aag2 (Aag2.Wol) cells without (Sup) and with filtration (0.2 µm) (F-Sup). The blot was probed with anti-WSP antibodies. (**B**) Western blot analysis of the *Wolbachia* pellet and the cytoplasmic supernatant with and without filtration, probed with anti-FtsZ antibodies. FtsZ is a bacterial cellular protein that is not known to be secreted.

Previously, the secretion of WSP into the host cytoplasm was shown only in nematodes (19). To determine if that is also the case in mosquito cells, we separated the host cell’s soluble fraction as the cytoplasmic fraction to analyze it with antibodies to *Wolbachia* proteins. This involved breaking Aag2.*w*MelPop cells by sonication, subjecting the lysate to high-speed centrifugation to precipitate *Wolbachia* and cell debris (pellet), and collecting the supernatant as the cytoplasmic fraction. Part of the supernatant was also filtered (0.2 µm) to exclude any potential *Wolbachia* particles. Aag2 cells were also treated similarly to use as control. Western blot analysis of the supernatant and filtered supernatant from the *Wolbachia*-infected cells showed the presence of the WSP recognized by an anti-WSP antibody; however, the protein was not detected in control Aag2 cells (Fig. 1A). Furthermore, the pellet, the supernatant, and the filtered supernatant from *Wolbachia*-infected cells, with and without DENV infection, were subjected to Western blot analysis using an anti-FtsZ antibody. This protein is not known to be a secreted bacterial protein. The FtsZ band was only found in the pellet (containing *Wolbachia*) and not in the supernatant or the filtered supernatant (cytoplasmic fractions) (Fig. 1B). These results suggested that similar to nematodes, the WSP is secreted into the cytoplasm in mosquito cells.

### *Wolbachia* WSP interacts with cellular as well as *Wolbachia* proteins

**Fig 2:**
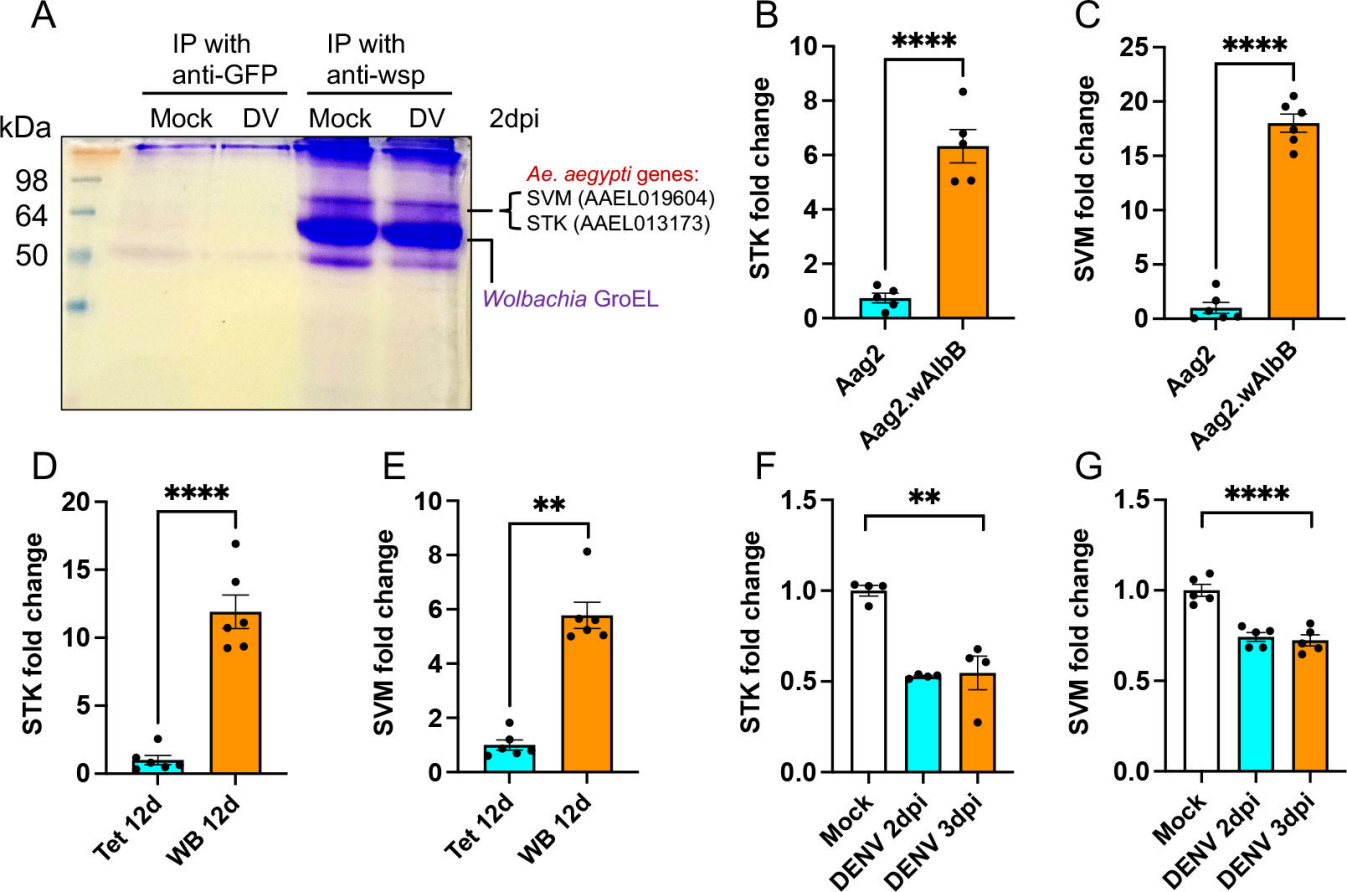
*Wolbachia* WSP interacts with cellular proteins. (**A**) Proteins eluted from IP with anti-GFP and anti-WSP antibodies run on polyacrylamide gel and Coomassie stained. Protein sizes are indicated by a pre-stained protein molecular weight ladder. Reverse transcription quantitative PCR analysis of *STK* and *SVM* expressions in (B and C) Aag2 and Aag2.*w*AlbB cell lines, (**D and E**) 12-day-old female mosquitoes transinfected with *w*AlbB (WB) or tetracycline-treated (Tet), and (**F and G**) DENV-infected Aag2.*w*AlbB cells. *t*-test was used for pair-wise comparisons in panels B–E and one-way analysis of variance for panels F and G with Tukey’s post hoc comparisons test analysis. The error bars indicate the standard error of the mean of three or more biological replicates. **P < 0.01, ****P < 0.0001.

To find out the possible interaction of *Wolbachia* WSP with cellular and *Wolbachia* proteins, we used anti-WSP antisera specific to *w*MelPop’s WSP in IP using *Wolbachia*-infected Aag2 cells. Anti-GFP was used as a negative control. Coomassie-stained gel showed distinct bands that appeared only with anti-WSP IP (Fig. 2A). Mass spectrometry analysis of the purified peptides from the bands (separately eluted) at 64 and 50 kDa revealed *Ae. aegypti* (cellular) and *Wolbachia* proteins, respectively. The identified cellular proteins were serine-threonine kinase (STK) (AAEL013173) and synaptic vesicle membrane (SVM) protein (AAEL019604). At the same time, GroEL was the only *Wolbachia* protein found, which plays a role as a chaperonin.

Next, we investigated the expression of *STK* and *SVM* in an *Ae. aegypti* cell line and in mosquitoes with and without *Wolbachia* infection using *w*AlbB-infected cells and mosquitoes due to the availability of the *w*AlbB (WB)-infected mosquitoes to us. Reverse transcription quantitative PCR (RT-qPCR) analysis showed significant upregulation of both *STK* and *SVM* transcripts in *Wolbachia*-infected cells compared to Aag2 cells (*t*-test, *P* < 0.0001) (Fig. 2B and C). A similar pattern of expression was also observed in WB mosquitoes compared to their controls (*t*-test, *P* < 0.0001; Mann-Whitney *P* = 0.0022) (Fig. 2D and E). In this experiment, tetracycline-treated or WB mosquitoes were collected at 12 days after emergence for RNA extraction. Significant induction of both genes in *Wolbachia*-infected cell line and mosquitoes indicates their potential importance in *Wolbachia*-host interaction. Furthermore, we looked for a correlation between the expression of *STK* and *SVM* with DENV infection in *Wolbachia*-infected Aag2 cells. Interestingly, both genes showed significant downregulation at 2 and 3 days post-infection (dpi) compared to uninfected mock samples (Kruskal-Willis and one-way analysis of variance [ANOVA]; *P* = 0.0043 and *P* = 0.0001, respectively) (Fig. 2F and G). These results highlight the potential role of *STK* and *SVM* in DENV infection in *Wolbachia*-infected cells.

### RNAi silencing of *STK* but not *SVM* inhibits DENV replication in the Aag2 cell line

**Fig 3:**
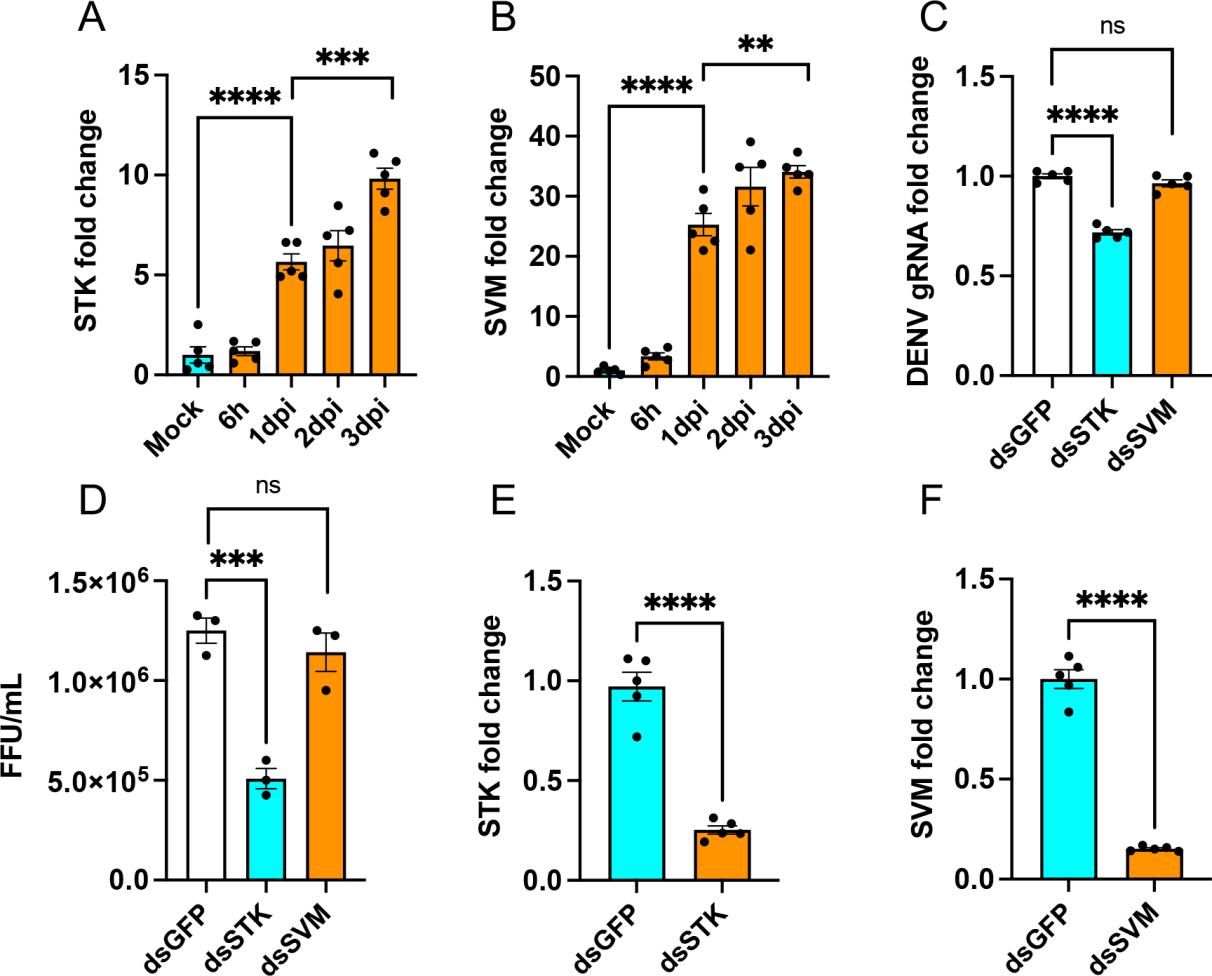
Differential expression and RNAi silencing of *STK* and *SVM* in Aag2 cells. Expression analysis of (**A**) *STK* and (**B**) *SVM* following DENV infection at different time points in Aag2 cells. (**C**) RT-qPCR analysis of RNA isolated from Aag2 cells transfected with ds*GFP*, ds*STK*, or dsSVM followed by DENV infection (1 multiplicity of infection) collected at 3 dpi to measure DENV gRNA levels. (**D**) Supernatants collected from the RNAi experiment were used in FFA to quantify DENV virions. (**E and F**) Confirmation of silencing of (**E**) *STK* and (**F**) *SVM* compared to control ds*GFP*-transfected cells. The error bars indicate the standard error of the mean of three or more biological replicates. One-way ANOVA was used to compare treatments for panels A–D with Tukey’s post hoc comparisons test analysis. *t*-test was carried out for pair-wise comparison of treatments in panels E and F. **P < 0.01, ***P < 0.001, ****P < 0.0001. FFU, focus forming unit; ns, not significant.

We were interested in investigating the role of *STK* and *SVM* in DENV infection in the absence of *Wolbachia*. First, we conducted a time course expression analysis of both genes in DENV-infected Aag2 cells. We observed significant upregulation of *STK* (one-way ANOVA, *P* < 0.0001) and *SVM* (*P* < 0.0001) from 1 to 3 dpi compared to uninfected mock or 6 h post-infection (Fig. 3A and B). That suggested the potential role of both genes in DENV replication in Aag2 cells. Next, both genes were silenced by *in vitro* synthesis of 450- to 500-bp dsRNAs of *STK* and *SVM*, which were transfected into Aag2 cells. The transfected cells were subsequently infected with DENV, and genomic RNA (gRNA) replication was analyzed in samples collected at 3 dpi. RT-qPCR analysis showed a significant reduction in DENV gRNA replication with ds*STK* (one-way ANOVA, *P* < 0.0001) but non-significant (*P* = 0.2361) with ds*SVM* when compared to ds*GFP* (Fig. 3C). To verify these results, we performed focus-forming assay (FFA) by using supernatants collected from the RNAi experiment. FFA also revealed a significant reduction of DENV virions with ds*STK* (one-way ANOVA, *P* = 0.0009) but not with ds*SVM* (*P* = 0.572). The efficient silencing of both genes (*t*-test, STK 74%, *P* < 0.0001, and SVM 85%, *P* < 0.0001) was confirmed by RT-qPCR (Fig. 3E and F). These results suggested that *STK* potentially plays an important role in DENV replication and could be considered a pro-viral gene. Since no effect on DENV gRNA replication and virions was seen in ds*SVM*-treated cells, we focused only on *STK* for further experiments.

### Silencing *STK* further reduces DENV replication in *Wolbachia*-infected cells

**Fig 4:**
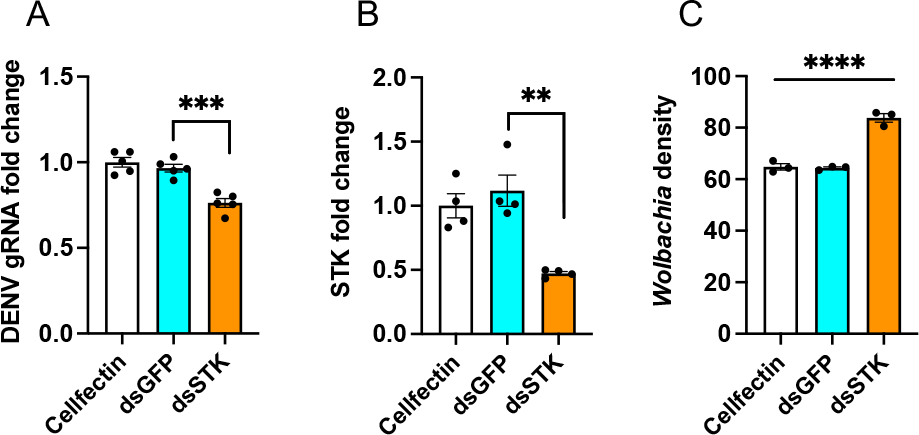
Effect of RNAi silencing of *STK* in Aag2.*w*AlbB cells on DENV and *Wolbachia* density. (**A**) RT-qPCR analysis of RNA extracted from *Wolbachia*-infected cells transfected with Cellfectin, ds*GFP*, or ds*STK* followed by DENV infection at 1 MOI for 3 days to assess DENV gRNA levels. (**B**) Confirmation of silencing of *STK* compared to control ds*GFP*-transfected cells. (**C**) qPCR analysis of total genomic DNA extracted from cells treated as in panel **A** using specific primers to *RPS17* and *WSP* genes. One-way ANOVA was used to compare treatments with Tukey’s post hoc comparisons test analysis. The error bars indicate the standard error of the mean of three or more biological replicates. **P < 0.01, ***P < 0.001, ****P < 0.0001.

Significant upregulation of *STK* in *Wolbachia*-infected Aag2 compared to uninfected Aag2 cells led us to investigate any possible effect on *Wolbachia* or DENV after RNAi silencing. For RNAi, *Wolbachia*-infected Aag2 cells were transfected with ds*STK*, while ds*GFP* was used as a negative control. Three days after transfection, cells were infected with DENV at a multiplicity of infection (MOI) of 1. RT-qPCR analysis of cells collected at 3 dpi showed a significant reduction in DENV gRNA levels after *STK* silencing (one-way ANOVA, *P* = 0.0003) compared to Cellfectin or ds*GFP*-transfected cells (Fig. 4A). Silencing of *STK* was also confirmed (57% relative to ds*GFP*; one-way ANOVA, *P* = 0.0017) (Fig. 4B). The results in *Wolbachia*-infected Aag2 cells are consistent with those in Aag2 cells (Fig. 3C and D). Of note, these assays were not done at the same time, but the conditions of the assays were identical. To examine if *STK* silencing affected *Wolbachia* density, we used gDNA from the same experiment and conducted quantitative PCR (qPCR) using *WSP* primers. The results showed a significant increase in *Wolbachia* density in ds*STK*-transfected cells (one-way ANOVA, *P* < 0.0001) (Fig. 4C).

### Induction of *STK* by RNA activation in *Wolbachia*-infected and uninfected Aag2 cells enhances DENV replication

**Fig 5:**
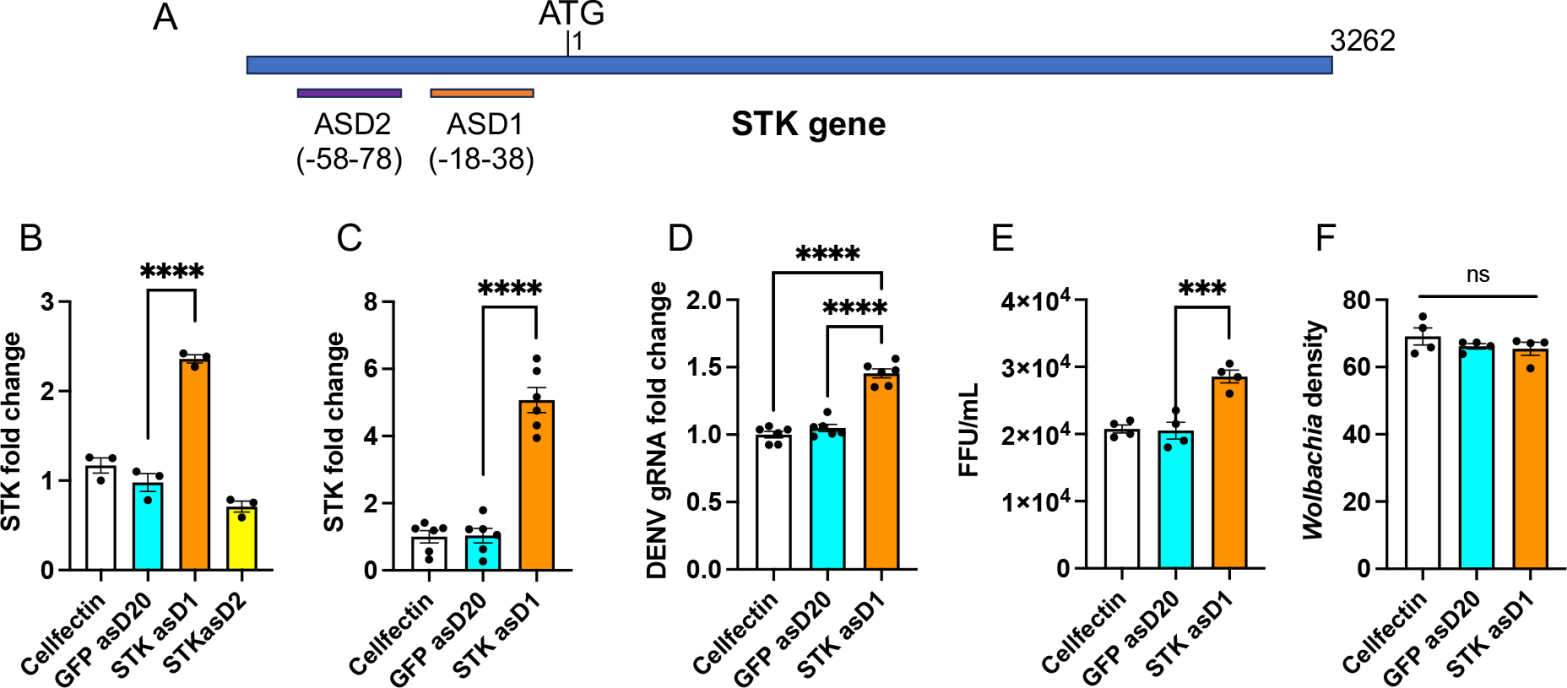
RNA activation to induce *STK* expression in Aag2.*w*AlbB cells. (**A**) Diagram showing the design of two 20-nt antisense DNAs (asDs) asD1 and asD2 in the promoter region of the *STK* gene. Twenty-nucleotide asD to GFP was used as control. (**B**) Confirmation of induction of the *STK* transcript levels in *Wolbachia*-infected cells transfected with asDs after 2 days of transfection. (**C**) Confirmation of the induction of *STK* in asD1-transfected and DENV-infected cells at 5 dpi. (**D**) RT-qPCR analysis to examine the effect of *STK* induction on DENV gRNA levels. (**E**) Supernatants taken from panel **D **were used for FFA to quantify DENV virions. (**F**) qPCR analysis of total genomic DNA extracted from the experiment described in (**D**) using specific primers to *RPS17* and *WSP* genes. One-way ANOVA was used to compare treatments with Tukey’s post hoc comparisons test analysis. The error bars indicate the standard error of the mean of three or more biological replicates. ***P < 0.001, ****P < 0.0001. FFU, focus forming unit; ns, not significant.

Since DENV infection in *Wolbachia*-infected cells led to significant downregulation of *STK*, we were keen to overexpress the gene and see the effect on DENV in these cells. For overexpression of *STK*, we utilized RNA activation by targeting the gene’s promoter region based on our previous study (25). We designed two different oligos (20 mer) in antisense at −18 to −38 (*STK* asD1) and −58 to −78 (*STK* asD2) positions of the *STK* promoter relative to the ATG start codon (Fig. 5A). To examine the induction of *STK* using the asDs, they were separately transfected into *Wolbachia* infected. GFP asD20 was used as a negative control. Cells were then collected 48 h after transfection. RT-qPCR analysis showed significant induction of *STK* with only *STK* asD1 (2.4-fold; one-way ANOVA, *P* < 0.0001), but not *STK* asD2, when compared to GFP asD20 (Fig. 5B). Our next question was whether asD1-mediated *STK* induction has any effect on DENV replication. For this, *Wolbachia*-infected cells were transfected with *STK* asD1, infected with DENV, and collected at 3 dpi. Significant induction of *STK* was observed in these cells (about fivefold; one-way ANOVA, *P* < 0.0001) that led to significant increases (*P* < 0.0001) in DENV gRNA (Fig. 5C and D). This effect on DENV replication was also confirmed by focus-forming assay, which showed a significantly higher number of virions (one-way ANOVA, *P* = 0.0006) in *STK*-induced cells (Fig. 5E). However, there was no change in *Wolbachia* density (Kruskal-Wallis test, *P* = 0.8147) after induction of the *STK* gene in the same transfection experiment (Fig. 5F).

**Fig 6:**
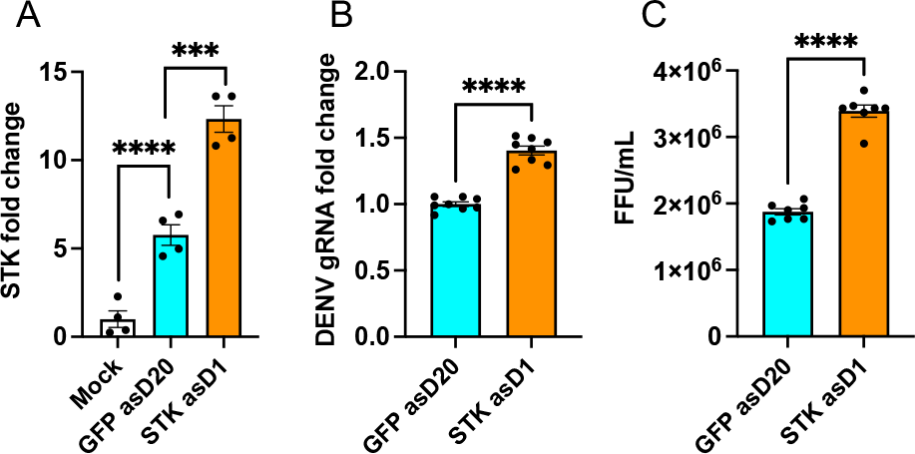
RNA activation to induce *STK* expression in Aag2 cells. (**A**) Confirmation of the induction of the *STK* expression in Aag2 cells transfected with *STK* asD1 and infected with DENV (1 MOI). Mock cells were uninfected. Twenty-nucleotide asD to *GFP* was used as control. (**B**) RT-qPCR analysis to assess the effect of *STK* induction on DENV genomic RNA levels. (**C**) Supernatants collected from panel **B** were used for FFA to quantify DENV virions. One-way ANOVA was used to compare treatments with Tukey’s post hoc comparisons test analysis for panel **A, **and a *t*-test was carried out for pair-wise comparison of treatments in panels B and C. The error bars indicate the standard error of the mean of three or more biological replicates. ***P < 0.001, ****P < 0.0001. FFU, focus forming unit.

Similar RNA activation of *STK* was also conducted in Aag2 cells, which showed significant induction of the *STK* transcripts (one-way ANOVA, *P* < 0.0001) (Fig. 6A) as well as an increase in DENV replication (*t*-test, *P* < 0.0001 for gRNA and virions) in *STK* asD1-transfected cells compared to GFP asD20-transfected cells (Fig. 6B and C). These results suggest that induction of the *STK* expression enhances DENV replication as a pro-viral gene.

### RNAi silencing of *STK* reduces DENV replication in *Ae. aegypti* mosquitoes

**Fig 7:**
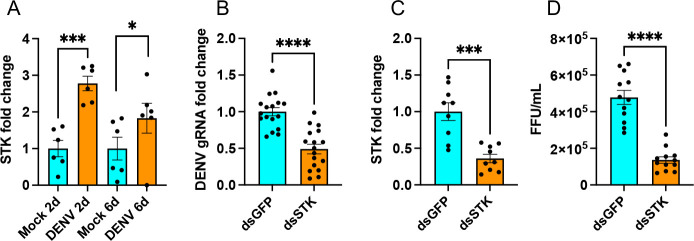
RNAi silencing of *STK* in *Ae. aegypti* mosquitoes and the effect on DENV replication. (**A**) Expression analysis of *STK* in uninfected and DENV-infected mosquitoes at 2 and 6 days following DENV infection. Total RNA was extracted from single mosquitoes and subjected to RT-qPCR. (**B**) RT-qPCR analysis of DENV gRNA levels in mosquitoes injected with ds*STK* or ds*GFP* and infected with DENV and collected at 4 dpi. (**C**) Confirmation of silencing of *STK* in dsRNA-injected mosquitoes from panel **B**. (**D**) Quantification of DENV virions in infected mosquitoes previously injected with ds*GFP* or ds*STK* using FFA. *t*-test was carried out for pair-wise comparison of treatments. The error bars indicate the standard error of the mean of biological replicates. *P < 0.05, ***P < 0.001, ****P < 0.0001. FFU, focus forming unit.

Considering the reduction in DENV replication in mosquito cell lines when *STK* was silenced, we were interested in exploring the effect of *STK* silencing on DENV replication in mosquitoes. Similar to DENV-infected Aag2 cells (Fig. 3A), we found significant upregulation of *STK* transcripts in DENV-infected mosquitoes at 2 and 6 dpi. This upregulation was more pronounced at 2 dpi (*t*-test, *P* = 0.0001) than at 6 dpi (*t*-test, *P* = 0.0143) when compared to their respective uninfected samples (Fig. 7A). RT-qPCR analysis of dsRNA-injected mosquitoes, which were orally inoculated with 10^7^/mL DENV, showed significant reductions in DENV replication in *STK*-silenced mosquitoes at 4 dpi (one-way ANOVA, *P* = 0.0001) compared to control ds*GFP*-injected mosquitoes (Fig. 7B). Confirmation of silencing of *STK* also showed significant knockdown of the gene’s transcripts (64%; one-way ANOVA, *P* = 0.0002) (Fig. 7C). In a repeat experiment, we quantified DENV virions in mosquitoes collected at 5 dpi using FFA. Results also demonstrated significant reductions (*t*-test, *P* < 0.0001) in the virus titer in mosquitoes injected with ds*STK* compared to ds*GFP*-injected mosquitoes (Fig. 7D). These results suggested that *STK* silencing led to reduced DENV replication in mosquitoes, as was observed in the Aag2 cell line.

### Expression of *STK* in *Wolbachia*-infected and uninfected *Ae. aegypti* mosquitoes, when infected with DENV

**Fig 8:**
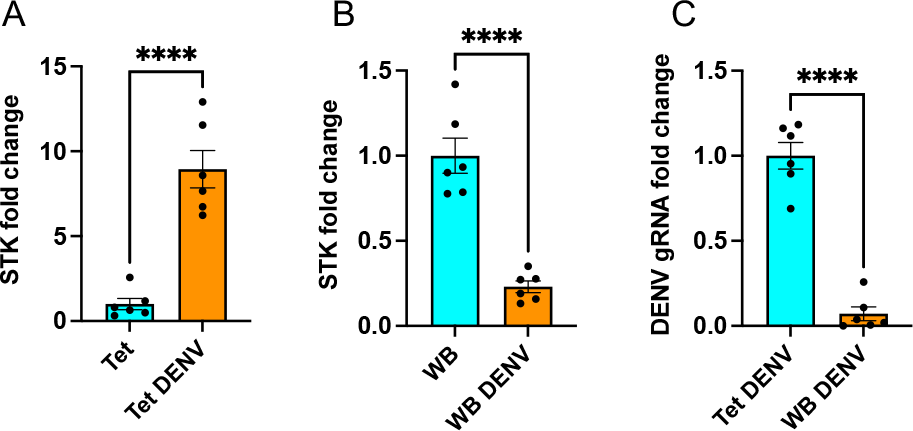
The expression levels of *STK* in *Wolbachia*-infected and uninfected mosquitoes when infected with DENV. RT-qPCR analysis of individual (**A**) Tet and (**B**) WB mosquitoes fed on DENV (10^7^ /mL) and collected at 3 dpi. (**C**) DENV gRNA fold changes in Tet and WB mosquitoes infected with DENV for 3 days. Unpaired *t*-test was carried out for comparison of treatments. The error bars indicate the standard error of the mean of six biological replicates. ****P < 0.0001. gRNA, genomic RNA.

Considering we observed the induction of *STK* in DENV-infected Aag2 cells (Fig. 3A) and its suppression in DENV-infected Aag2 cells infected with *Wolbachia* (Fig. 2F), we examined the *STK* expression levels in Tet and WB mosquitoes. For this, 4-day-old female mosquitoes were orally infected with DENV and collected at 3 dpi. Similarly, we found significant induction (*t*-test, *P* < 0.0001) of *STK* in Tet mosquitoes following DENV replication (Fig. 8A) but a significant reduction (*t*-test, *P* < 0.0001) in its expression in DENV-infected WB mosquitoes (Fig. 8B). To confirm the virus blocking effect in the mosquitoes, DENV gRNA levels were compared between Tet and WB DENV-infected mosquitoes. In WB mosquitoes, DENV gRNA levels were significantly less (*t*-test, *P* < 0.0001) than those in Tet mosquitoes (Fig. 8C). Considering the *STK* levels were already reduced by 75% in WB mosquitoes when infected with DENV (Fig. 8B), we did not attempt to silence the *STK* gene in WB mosquitoes to assess the effect on DENV replication.

### Overexpression of the WSP reduces DENV replication

**Fig 9:**
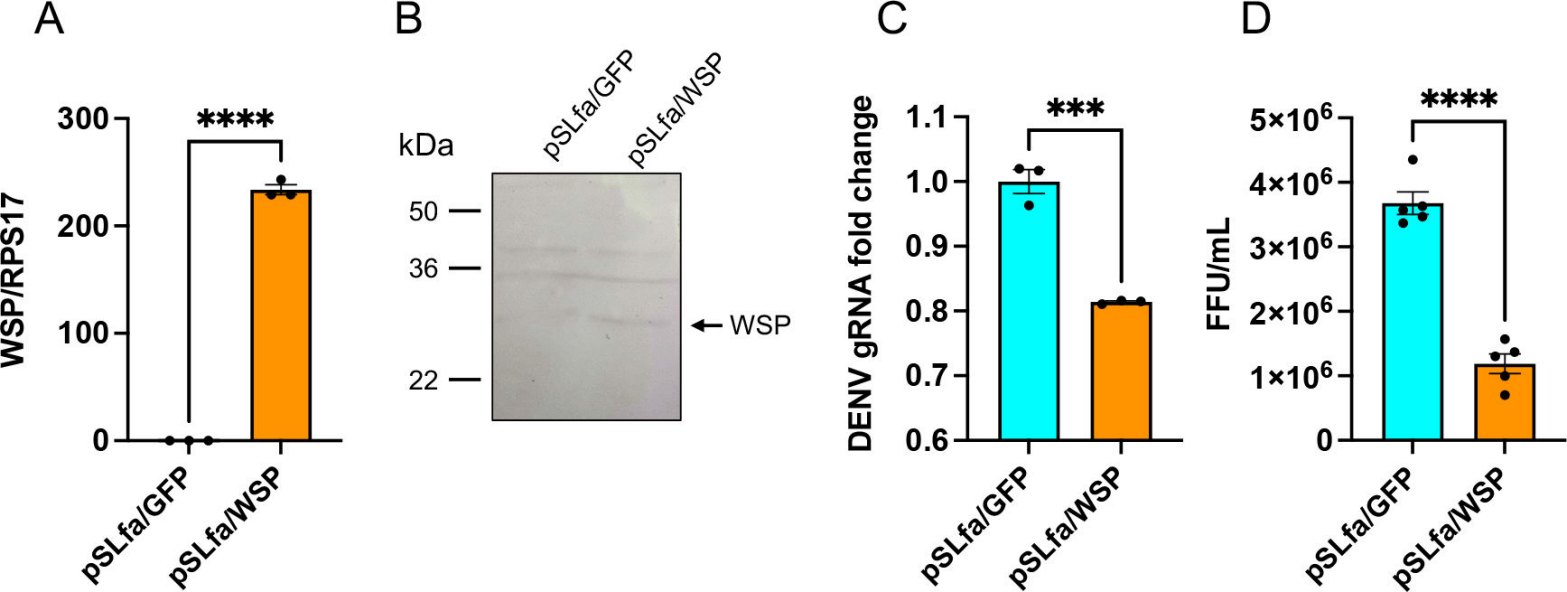
The effect of overexpression of WSP in Aag2 cells on DENV replication. Aag2 cells without *Wolbachia* infection were transfected with pSLfa-WSP or pSLfa-GFP as a control. Cells were then infected with 1-MOI DENV. (**A**) qPCR analysis of cells transfected with pSLfa-GFP and pSLfa-WSP. (**B**) Western blot analysis of the transfected cells at 3 days post-transfection using the anti-WSP antibody. (**C**) RT-qPCR analysis of RNA extracted from the transfected cells collected 4 days after DENV infection. (**D**) Supernatants collected from panel **C **were used for FFA to quantify DENV virions. *t*-test was carried out for unpaired comparison of treatments. The error bars indicate the standard error of the mean of biological replicates. ***P < 0.001, ****P < 0.0001. FFU, focus forming unit.

Considering WSP is a secreted protein that interacts with STK, we hypothesized that by binding to STK, WSP may reduce the amount of available STK, leading to less DENV replication. We exogenously overexpressed WSP in Aag2 cells without *Wolbachia* infection to test this hypothesis. Following confirmation of overexpression of the gene and the protein (Fig. 9A and B), the cells were infected with 1 MOI of DENV. RT-qPCR analysis of cells at 4 dpi revealed a statistically significant reduction (*t*-test, *P* < 0.0005) in DENV gRNA levels (Fig. 9C). This reduction (*t*-test, *P* < 0.0001) was also confirmed by titration of virions (Fig. 9D). The results suggest that the secreted WSP may sequester STK and negatively affect DENV replication.

## DISCUSSION

*Wolbachia* restricts flavivirus replication and transmission in mosquitoes. This antiviral feature suggests that *Wolbachia* has evolved mechanisms to manipulate certain host cellular pathways. Interaction of *Wolbachia* and host proteins could strengthen endosymbiosis (26); however, concerning antiviral activity, this aspect has been less explored. We carried out a co-IP experiment to identify proteins interacting with WSP, based on its abundant expression in cells as *Wolbachia*’s surface protein. In addition, a previous study revealed that *Wolbachia* can secrete WSP into nematode tissues (19). Therefore, we hypothesized that if WSP is secreted into mosquito cells, it might interact with host proteins.

Purification of cellular fractions of *Wolbachia*-infected cells confirmed that WSP is also secreted into the host cytoplasm in mosquito cells. In our co-IP assay conducted using Aag2.*w*MelPop cells and anti-WSP antibodies, two host proteins (STK and SVM) and one *Wolbachia* protein (GroEL) were found to bind to *w*MelPop WSP. Interestingly, expression analysis based on RT-qPCRs showed induction of both *STK* and *SVM* in *Wolbachia*, as well as DENV-infected Aag2 cells and mosquitoes. However, both genes were downregulated upon DENV infection in Aag2.*w*AlbB cells. The abrupt downregulation of both genes by *Wolbachia* after DENV infection hints at the potential roles of these genes in DENV infection and their suppression by *Wolbachia* to enhance antiviral capability. In addition, RNAi knockdown of *STK*, but not *SVM*, in Aag2 cells resulted in a significant decrease in DENV replication, which indicates that STK might have a pro-viral role. A similar knockdown effect of *STK* on DENV replication was also observed *in vivo* after DENV was introduced to the ds*STK*-injected mosquitoes. These results further strengthen evidence of a putative pro-viral role of STK.

The primary function of STK is to catalyze the transfer of a phosphate from ATP to a serine or threonine amino acid residue of the protein substrate (27). In our analysis, the upregulation of *STK* transcripts in *Wolbachia* cells in the absence of virus infection hints at the importance of the protein in the normal life cycle of the bacterium, which is supported by a previous finding that eukaryotic STKs are associated with the regulation of bacterial virulence and cell division (28). Concerning virus infection, there are several reports available indicating that serine-threonine phosphorylation is a conserved feature across flaviviruses. Phosphorylation of the NS5 proteins of hepatitis C virus (HCV), DENV, West Nile virus, and YFV are well-studied examples (29, 30). Phosphorylation of NS5B has also been shown to affect the replicon activity of HCV (31). Phosphorylation of the NS5 protein has been demonstrated in cells infected with DENV serotype 2 (32) and in extracts of cells infected with tick-borne encephalitis virus (33). Other studies have revealed that NS5s of DENV-2 and HCV are phosphorylated, preferentially on serine residues (29, 34). Furthermore, NS5 phosphorylation may regulate viral replication and/or the expression of host genes, among other possibilities (32). Moreover, in poxvirus, STK induces proteins associated with the assembly of new virions in infected cells (35). Another type of STK (SGK1) is a host factor, which is involved in viral infection and promotes the replication of the influenza virus (36). SGK1 also facilitates the entry of human immunodeficiency virus type 1 into host cells (37) and stimulates the productive infection of bovine herpesvirus 1 and herpes simplex virus 1 (38).

During the last decade, transcriptional activation has been widely utilized to achieve higher protein expression of endogenous genes using several types of RNAs such as natural antisense transcripts, promoter RNAs, enhancer RNAs, and antisense oligonucleotides (ASO) (39). Previously, we and others have reported RNA activation by using antisense RNA to the promoter region of genes (25, 40, 41). We further confirmed the pro-viral activity of STK by inducing its expression in Aag2 as well as Aag2.*w*AlbB cell line by RNA activation using an ASO, which led to significantly higher levels of DENV gRNA as well as virions. We observed that after inducing *STK*, the DENV gRNA levels were comparably greater in Aag2.*w*AlbB cells than in Aag2 cells. The possible reason is that STK levels are higher already in DENV-infected Aag2 cells, and further induction of the gene has a minimal advantage.

In summary, we showed that *Wolbachia* surface protein is a secreted protein in mosquito cells, where it interacts with the *Ae. aegypti* STK protein. The gene was induced in both *Wolbachia*-infected and DENV-infected cells; however, when combined, *STK* was downregulated. Silencing *STK* using RNAi reduced DENV replication both *in vitro* and *in vivo*, while its induction through RNA activation increased DENV replication. This suggests that STK plays a pro-viral role in DENV replication in mosquitoes. Moreover, suppressing *STK* in *Wolbachia*-infected cells infected with DENV may hinder the virus’s replication. Additionally, the binding of secreted WSP to STK could sequester the pro-viral protein, making it less available to DENV. Overall, these results enhance our understanding of the mosquito-*Wolbachia*-DENV interaction. However, limitations of this study include the lack of access to mosquitoes with other *Wolbachia* strains and appropriate containment facilities to test other arboviruses, such as Chikungunya virus. Consequently, the findings from the *w*AlbB strain and DENV may not apply to other strains or arboviruses.

## MATERIALS AND METHODS

### Mosquitoes and mosquito cell lines

*Aedes aegypti* mosquitoes were maintained at 28°C, a 12-h light/12-h dark cycle, and 70% relative humidity on 10% sugar solution in water. Female mosquitoes were artificially fed on human blood obtained from the Australian Red Cross using glass feeders. *w*AlbB-transinfected and tetracycline-cured *Ae. aegypti* mosquitoes (42) were used for analyzing mosquito gene expressions.

*Aedes aegypti* cell line Aag2 and Aag2 cells infected with two *Wolbachia* strains, *w*MelPop (Aag2.*w*MelPop) (43) and *w*AlbB (Aag2.*w*AlbB) (44), were used in this study. These cell lines were maintained as cell monolayers in a flask in a 1:1 mixture of Mitsuhashi–Maramorosch (HIMEDIA) and Schneider’s Insect Medium (Invitrogen), supplemented with 10% fetal bovine serum (FBS) at 27°C. For infection, cells were infected with DENV-2 (ET-300 strain) at an MOI of 1.

For mosquito infections, 2-day-old female mosquitoes were injected with 1-µg dsRNA (see below) with a Nanoject III (Drummond) and pulled glass needles. Two days after injections, mosquitoes starved for 24 h were orally fed on human blood donated by the Red Cross containing 1 × 10^7^/mL DENV-2 (ET-300 strain) using glass feeders. Those mosquitoes that took a blood meal were collected and maintained for 4 days at 27°C and 70% relative humidity. Subsequently, total RNA was extracted from individual mosquitoes and subjected to RT-qPCR (see below). In total, 17 individual mosquitoes were analyzed individually as biological replicates for the analysis.

### Isolation of the host cell cytoplasmic fraction to examine secretion of WSP

To isolate the host cell cytoplasmic fraction, we utilized a protocol used for *Wolbachia* purification with some modifications (45). Briefly, Aag2 (as control) and Aag2.*w*MelPop cells (with and without DENV infection) were grown to confluency in cell culture flasks. Cells were resuspended in the medium and centrifuged at 1,000 × *g* for 10 min at 4°C. Following the removal of the medium, the pellet was resuspended in 5-mL SPG buffer (218-mM sucrose, 3.8-mM KH_2_PO_4_, 7.2-mM K_2_HPO_4_, 4.9-mM L-glutamate, pH 7.2) and centrifuged at 1,000 × *g* for 10 min at 4°C to pellet the cells. After removing the supernatant, the pellet was resuspended in 10-mL SPG buffer and sonicated on ice with a Q125 sonicator (Qsonica) using 3 × 10 s bursts at 22% power. The lysate was centrifuged at 13,800 × *g* for 20 min at 4°C to collect *Wolbachia* and cell debris (pellet). The supernatant was collected as the *Wolbachia*-free cytoplasmic fraction (Sup). A fraction of the supernatant was also passed through a 0.2-M filter to remove any potential *Wolbachia* particles (F-Sup). The fractions were analyzed by Western blotting. The purification method was repeated at least three times with reproducible results.

### Overexpression of WSP

The full-length open reading frame for *w*MelPop WSP was cloned into the pSLfa vector (Addgene) to generate pSLfa-WSP. The plasmid was subjected to sequencing to confirm the coding region is intact. Subsequently, 2-µg plasmid was transfected into Aag2 cells (without *Wolbachia* infection) using Cellfectin II reagent (Invitrogen) according to the manufacturer’s instructions. A pSLfa-GFP plasmid was used as a control. Two days post-transfection, the cells were collected for Western blot analysis to confirm overexpression of the protein. Transfected cells were also infected with 1-MOI DENV and collected at 4 dpi for RNA extraction and assessment of viral gRNA replication.

### Western blot analysis

Protein samples were separated on a 12% SDS-PAGE and transferred onto a nitrocellulose membrane. After blocking, the membrane was incubated with anti-WSP (1:2,000) or anti-FtsZ (1:20,000; Abcam) polyclonal antibodies. The primary antibodies were detected using anti-rabbit antibodies conjugated with alkaline phosphatase (1:10,000; Sigma), followed by developing the blots with NBT/BCIP (Thermo Fisher Scientific).

### IP

To find the interaction of cellular or *Wolbachia* proteins with WSP, IP was conducted by using an anti-WSP polyclonal antibody, while anti-GFP was used as a negative control. Aag2.*w*MelPop cells were lysed in 1× RIPA Cell Lysis buffer (Thermo Fisher Scientific), and the lysate was incubated with the antibody for 2 h at 4°C with gentle shaking. Protein A beads (50 µL) were added to the antibody-antigen lysate and incubated on ice for 1 h. Beads were collected by centrifugation at 10,000 × *g* for 1 min at 4°C followed by four washes with IP wash buffer (NaCl 150 mM, EDTA 1 mM, Triton X-100 1%, and Tris [pH 7.4] 10 mM). The captured antigen was eluted with 1× RIPA buffer and run on 12% SDS-PAGE protein gel. Distinct protein bands appeared after Coomassie staining and destaining. The distinct gel protein bands at 50 and 64 kDa were cut and purified for mass spectrometry using a protocol previously described (46). Peptides were digested with trypsin and desalted using 0.6-µL C_18_ ZipTip (Merck) according to the manufacturer’s protocol. Eluted peptides were analyzed on a TripleTOf 5600 instrument (SCIEX) using a Nanospray III interface. Peptides and proteins were identified by ProteinPilot v.5.0.1 (SCIEX) using the *Ae. aegypti* proteins (AaegL5.2) as well as *Wolbachia* proteins. The identified proteins were analyzed for further studies.

### RNA extraction and RT-qPCR

Total RNA extraction was carried out from cell pellets using Qiazol (Qiagen) according to the manufacturer’s instructions. For mosquitoes, they were singly homogenized in 500-µL Qiazol with two beads (Qiagen) using the TissueLyser II (Qiagen) at 30 cycles/s for 90 s. RNA was then extracted according to the manufacturer’s instructions for Qiazol. DNase treatment was done using Turbo DNase (Ambion), and RNA was quantified using an Epoch spectrophotometer (BioTek). cDNA synthesis was done using M-MuLV reverse transcriptase (New England Biolabs) according to the manufacturer’s instructions. For qPCR reaction using QuantiFast SYBR Green PCR Kit (Qiagen), 10 times dilution of cDNA was used. qPCR was run on a Rotor-Gene Q machine (Qiagen), and PCR amplification and melt curve analysis for all products were performed according to the instructions. Primers used in this study are listed in Table 1. All qPCR experiments were run with at least three biological and two technical replicates.

**TABLE 1:** Primer sequences used in this study

Primer	Sequence
WALB WSP qF	ATCTTTTATGGCTGGTGGTGCT
WALB WSP qR	GGAGTGATAGGCATATCTTCAAT
STK ASD 1	TGTTGTCCGTGTCTGCTGTG
STK ASD 2	TGCCGCACCGCTGCTGCTTG
SVM qF	ACAAAGCCAAATCCGAACCC
SVM qR	CATGCTTTCACCCGGATCAG
SVM dsRNA F	TAATACGACTCACTATAGGGAGTGGACAAAGTATCGCCGA
SVM dsRNA R	TAATACGACTCACTATAGGGTCCTCCTTCTTTTCCGCACT
STK qF	TCGTTACCGCTGGAACTACA
STK qR	TTCATCACACGAATCTCCGC
STK dsRNA F	TAATACGACTCACTATAGGGAACATGTACCTGGTGCTGGA
STK dsRNA R	TAATACGACTCACTATAGGGTCCTGTTGATTGTCCGGTGA
GFP dsRNA F	TAATACGACTCACTATAGGGGGTGATGCAACATACGG
GFP dsRNA R	TAATACGACTCACTATAGGGGCAGATTGTGTGGACAG
DENV qF	GGTATGGTGGGCGCTACTA
DENV qR	GGTATGGTGGGCGCTACTA
RPS 17 qF	CACTCCGAGGTCCGTGGTAT
RPS 17 qR	GGACACTTCGGGCACGTAGT

### Determination of *Wolbachia* density

Genomic DNA was isolated from cells using EconoSpin silica membrane columns (Epoch Life Science) according to a previously described protocol (47). The relative density of *w*AlbB was quantified by qPCR using specific primers for the *wsp* gene and the *Ae. aegypti RPS17* gene for normalizing the data. Primer sequences can be found in Table 1. Three biological replicates with two technical replicates were used for density determinations.

### Focus-forming assay

Focus-forming assay was conducted using supernatants collected from transfection infection experiments or lysed mosquitoes. For mosquitoes, the body of each mosquito was homogenized in 500 µL of grinding medium (RPMI 1640, supplemented with 2% FBS, 1% pen-strep, 1% amphotericin B), followed by centrifugation at 7,600 × *g* for 5 min at 4°C. The supernatant (100 µL/well) was used to inoculate C6/36. The assay was conducted in C6/36 cells in 96-well plates according to a previously described method (48). For infection, 10 µL/well of supernatants was used, and plates were first kept on a rocker for 1 h at room temperature and then incubated at 37°C for an extra hour. At 3 dpi, cells were fixed in ice-cold 80% acetone in PBS for 20 min at −20°C and then dried overnight. Then, cells were blocked with 5% skim milk in phosphate-buffered saline with Tween (PBST) at 37°C for 30 min. This was followed by incubating cells with antisera specific to DENV-2 E protein, 4E11 (1:1,000) in 0.1% skim milk in PBST for 2 h at 37°C. After that, plates were washed three times with PBST followed by probing with secondary antibody IRDYE 800CW goat anti-human (1:2,500) for 1 h at 37°C. Plates were washed three times with PBST and dried as above and scanned for foci detection and counting by LI-COR Biosciences Odyssey Infrared Imaging System according to manual instructions. The titrations were repeated three times.

### *In vitro* dsRNA synthesis

We used MEGAscript T7 transcription kit (Invitrogen) for *in vitro* synthesis of dsRNA of target genes and control *GFP*. Gene-specific forward and reverse primers containing T7 promoter sequences were used for PCR amplification of products (500 nt), which were confirmed after sequencing. In the transcription reaction, 1 µg of purified PCR product and all the required ingredients were incubated at 37°C overnight. After DNase treatment, dsRNA was precipitated using lithium chloride, washed with 70% ethanol, and finally dissolved in nuclease-free water. The quantity and quality of dsRNA were measured using an Epoch spectrophotometer (BioTek) and by running it on 1% agarose gel. Primers used for dsRNA synthesis are listed in Table 1.

dsRNA was transfected into cells using the Cellfectin II transfection reagent (Invitrogen). Cells (5 × 10^5^) were seeded in 12-well plates and allowed to settle for 1 h. For each treatment, 2 µg of dsRNA was transfected into cells according to the manufacturer’s instructions.

### Quantification and statistical analysis

GraphPad Prism v.10 was used for all the statistical analyses and production of the graphs. For data that passed the normality test, *t*-test or one-way ANOVA with Tukey’s post hoc comparisons test was used to determine significance levels between two and three or more treatments, respectively. If data did not pass the normality test, non-parametric tests were used. More details are provided in the relevant figure legends or the text. qPCR data were analyzed using the relative expression ratio method [ratio = (*E*_target_)^ΔCP^_target(control – sample)_/(*E*_ref_)^ΔCP^_ref(control – sample)_] as described previously (49).

## Data Availability

The data sets generated and/or analyzed during the current study are available from the corresponding author.

## References

[1] Kaur R, Shropshire JD, Cross KL, Leigh B, Mansueto AJ, Stewart V, Bordenstein SR, Bordenstein SR. 2021. Living in the endosymbiotic world of Wolbachia: a centennial review. Cell Host Microbe 29:879–893. doi:10.1016/j.chom.2021.03.006 33945798 PMC8192442

[2] Weinert LA, Araujo-Jnr EV, Ahmed MZ, Welch JJ. 2015. The incidence of bacterial endosymbionts in terrestrial arthropods. Proc Biol Sci 282:20150249. doi:10.1098/rspb.2015.0249 25904667 PMC4424649

[3] Porter J, Sullivan W. 2023. The cellular lives of Wolbachia. Nat Rev Microbiol 21:750–766. doi:10.1038/s41579-023-00918-x 37430172

[4] Hedges LM, Brownlie JC, O’Neill SL, Johnson KN. 2008. Wolbachia and virus protection in insects. Science 322:702. doi:10.1126/science.1162418 18974344

[5] Teixeira L, Ferreira A, Ashburner M. 2008. The bacterial symbiont Wolbachia induces resistance to RNA viral infections in Drosophila melanogaster. PLoS Biol 6:e2. doi:10.1371/journal.pbio.1000002 PMC2605931 19222304

[6] Pacidônio EC, Caragata EP, Alves DM, Marques JT, Moreira LA. 2017. The impact of Wolbachia infection on the rate of vertical transmission of dengue virus in Brazilian Aedes aegypti. Parasit Vectors 10:296. doi:10.1186/s13071-017-2236-z 28623959 PMC5474007

[7] Ryan PA, Turley AP, Wilson G, Hurst TP, Retzki K, Brown-Kenyon J, Hodgson L, Kenny N, Cook H, Montgomery BL, Paton CJ, Ritchie SA, Hoffmann AA, Jewell NP, Tanamas SK, Anders KL, Simmons CP, O’Neill SL. 2019. Establishment of wMel Wolbachia in Aedes aegypti mosquitoes and reduction of local dengue transmission in Cairns and surrounding locations in northern Queensland, Australia. Gates Open Res 3:1547. doi:10.12688/gatesopenres.13061.2 31667465 PMC6801363

[8] Tantowijoyo W, Andari B, Arguni E, Budiwati N, Nurhayati I, Fitriana I, Ernesia I, Daniwijaya EW, Supriyati E, Yusdiana DH, Victorius M, Wardana DS, Ardiansyah H, Ahmad RA, Ryan PA, Simmons CP, Hoffmann AA, Rancès E, Turley AP, Johnson P, Utarini A, O’Neill SL. 2020. Stable establishment of wMel Wolbachia in Aedes aegypti populations in Yogyakarta, Indonesia. PLoS Negl Trop Dis 14:e0008157. doi:10.1371/journal.pntd.0008157 32302295 PMC7190183

[9] Bhattacharya T, Newton ILG, Hardy RW. 2020. Viral RNA is a target for Wolbachia-mediated pathogen blocking. PLoS Pathog 16:e1008513. doi:10.1371/journal.ppat.1008513 32555677 PMC7326284

[10] Hussain M, Zhang G, Leitner M, Hedges LM, Asgari S. 2023. Wolbachia RNase HI contributes to virus blocking in the mosquito Aedes aegypti. iScience 26:105836. doi:10.1016/j.isci.2022.105836 36636344 PMC9830209

[11] Thomas S, Verma J, Woolfit M, O’Neill SL. 2018. Wolbachia-mediated virus blocking in mosquito cells is dependent on XRN1-mediated viral RNA degradation and influenced by viral replication rate. PLoS Pathog 14:e1006879. doi:10.1371/journal.ppat.1006879 29494679 PMC5833283

[12] White PM, Serbus LR, Debec A, Codina A, Bray W, Guichet A, Lokey RS, Sullivan W. 2017. Reliance of Wolbachia on high rates of host proteolysis revealed by a genome-wide RNAi screen of Drosophila cells. Genetics 205:1473–1488. doi:10.1534/genetics.116.198903 28159754 PMC5378107

[13] Geoghegan V, Stainton K, Rainey SM, Ant TH, Dowle AA, Larson T, Hester S, Charles PD, Thomas B, Sinkins SP. 2017. Perturbed cholesterol and vesicular trafficking associated with dengue blocking in Wolbachia-infected Aedes aegypti cells. Nat Commun 8:526. doi:10.1038/s41467-017-00610-8 28904344 PMC5597582

[14] Rainey SM, Geoghegan V, Lefteri DA, Ant TH, Martinez J, McNamara CJ, Kamel W, de Laurent ZR, Castello A, Sinkins SP. 2023. Differences in proteome perturbations caused by the Wolbachia strain wAu suggest multiple mechanisms of Wolbachia-mediated antiviral activity. Sci Rep 13:11737. doi:10.1038/s41598-023-38127-4 37474590 PMC10359319

[15] Pan X, Zhou G, Wu J, Bian G, Lu P, Raikhel AS, Xi Z. 2012. Wolbachia induces reactive oxygen species (ROS)-dependent activation of the Toll pathway to control dengue virus in the mosquito Aedes aegypti. Proc Natl Acad Sci U S A 109:E23–E31. doi:10.1073/pnas.1116932108 22123956 PMC3252928

[16] Reyes JIL, Suzuki Y, Carvajal T, Muñoz MNM, Watanabe K. 2021. Intracellular interactions between arboviruses and wolbachia in Aedes aegypti. Front Cell Infect Microbiol 11:690087. doi:10.3389/fcimb.2021.690087 34249780 PMC8261290

[17] Martin M, López-Madrigal S, Newton ILG. 2024. The Wolbachia WalE1 effector alters Drosophila endocytosis. PLoS Pathog 20:e1011245. doi:10.1371/journal.ppat.1011245 38547310 PMC11003677

[18] Mills MK, McCabe LG, Rodrigue EM, Lechtreck KF, Starai VJ. 2023. Wbm0076, a candidate effector protein of the Wolbachia endosymbiont of Brugia malayi, disrupts eukaryotic actin dynamics. PLoS Pathog 19:e1010777. doi:10.1371/journal.ppat.1010777 36800397 PMC9980815

[19] Melnikow E, Xu S, Liu J, Bell AJ, Ghedin E, Unnasch TR, Lustigman S. 2013. A potential role for the interaction of Wolbachia surface proteins with the Brugia malayi glycolytic enzymes and cytoskeleton in maintenance of endosymbiosis. PLoS Negl Trop Dis 7:e2151. doi:10.1371/journal.pntd.0002151 23593519 PMC3617236

[20] Guabiraba R, Ryffel B. 2014. Dengue virus infection: current concepts in immune mechanisms and lessons from murine models. Immunology 141:143–156. doi:10.1111/imm.12188 24182427 PMC3904235

[21] Murray CL, Jones CT, Rice CM. 2008. Architects of assembly: roles of Flaviviridae non-structural proteins in virion morphogenesis. Nat Rev Microbiol 6:699–708. doi:10.1038/nrmicro1928 18587411 PMC2764292

[22] Shah PS, Link N, Jang GM, Sharp PP, Zhu T, Swaney DL, Johnson JR, Von Dollen J, Ramage HR, Satkamp L, et al.. 2018. Comparative flavivirus-host protein interaction mapping reveals mechanisms of dengue and Zika virus pathogenesis. Cell 175:1931–1945. doi:10.1016/j.cell.2018.11.028 30550790 PMC6474419

[23] Barrows NJ, Anglero-Rodriguez Y, Kim B, Jamison SF, Le Sommer C, McGee CE, Pearson JL, Dimopoulos G, Ascano M, Bradrick SS, Garcia-Blanco MA. 2019. Dual roles for the ER membrane protein complex in flavivirus infection: viral entry and protein biogenesis. Sci Rep 9:9711. doi:10.1038/s41598-019-45910-9 31273220 PMC6609633

[24] Hussain M, Bradshaw T, Lee M, Asgari S. 2022. The involvement of Atlastin in dengue virus and Wolbachia infection in Aedes aegypti and its regulation by aae-miR-989. Microbiol Spectr 10:e0225822. doi:10.1128/spectrum.02258-22 36165808 PMC9603060

[25] De Hayr L, Asad S, Hussain M, Asgari S. 2020. RNA activation in insects: the targeted activation of endogenous and exogenous genes. Insect Biochem Mol Biol 119:103325. doi:10.1016/j.ibmb.2020.103325 31978586

[26] Lindsey ARI. 2020. Sensing, signaling, and secretion: a review and analysis of systems for regulating host interaction in Wolbachia. Genes 11:813. doi:10.3390/genes11070813 32708808 PMC7397232

[27] Diallo A, Prigent C. 2011. The serine/threonine kinases that control cell cycle progression as therapeutic targets. Bull Cancer 98:1335–1345. doi:10.1684/bdc.2011.1467 22020767

[28] Pereira SFF, Goss L, Dworkin J. 2011. Eukaryote-like serine/threonine kinases and phosphatases in bacteria. Microbiol Mol Biol Rev 75:192–212. doi:10.1128/MMBR.00042-10 21372323 PMC3063355

[29] Kapoor M, Zhang L, Ramachandra M, Kusukawa J, Ebner KE, Padmanabhan R. 1995. Association between NS3 and NS5 proteins of dengue virus type 2 in the putative RNA replicase is linked to differential phosphorylation of NS5. J Biol Chem 270:19100–19106. doi:10.1074/jbc.270.32.19100 7642575

[30] Neddermann P, Quintavalle M, Di Pietro C, Clementi A, Cerretani M, Altamura S, Bartholomew L, De Francesco R. 2004. Reduction of hepatitis C virus NS5A hyperphosphorylation by selective inhibition of cellular kinases activates viral RNA replication in cell culture. J Virol 78:13306–13314. doi:10.1128/JVI.78.23.13306-13314.2004 15542681 PMC524975

[31] Kim S-J, Kim J-H, Kim Y-G, Lim H-S, Oh J-W. 2004. Protein kinase C-related kinase 2 regulates hepatitis C virus RNA polymerase function by phosphorylation. J Biol Chem 279:50031–50041. doi:10.1074/jbc.M408617200 15364941

[32] Reed KE, Gorbalenya AE, Rice CM. 1998. The NS5A/NS5 proteins of viruses from three genera of the family flaviviridae are phosphorylated by associated serine/threonine kinases. J Virol 72:6199–6206. doi:10.1128/JVI.72.7.6199-6206.1998 9621090 PMC110437

[33] Morozova OV, Tsekhanovskaya NA, Maksimova TG, Bachvalova VN, Matveeva VA. 1997. Phosphorylation of tick-borne encephalitis virus NS5 protein. Virus Res 49:9–15. doi:10.1016/s0168-1702(96)01433-5 9178492

[34] Kaneko T, Tanji Y, Satoh S, Hijikata M, Asabe S, Kimura K, Shimotohno K. 1994. Production of two phosphoproteins from the NS5A region of the hepatitis C viral genome. Biochem Biophys Res Commun 205:320–326. doi:10.1006/bbrc.1994.2667 7999043

[35] Johnston JB, Barrett JW, Chang W, Chung C-S, Zeng W, Masters J, Mann M, Wang F, Cao J, McFadden G. 2003. Role of the serine-threonine kinase PAK-1 in myxoma virus replication. J Virol 77:5877–5888. doi:10.1128/jvi.77.10.5877-5888.2003 12719581 PMC154029

[36] König R, Stertz S, Zhou Y, Inoue A, Hoffmann H-H, Bhattacharyya S, Alamares JG, Tscherne DM, Ortigoza MB, Liang Y, et al.. 2010. Human host factors required for influenza virus replication. Nature New Biol 463:813–817. doi:10.1038/nature08699 PMC2862546 20027183

[37] Rato S, Maia S, Brito PM, Resende L, Pereira CF, Moita C, Freitas RP, Moniz-Pereira J, Hacohen N, Moita LF, Goncalves J. 2010. Novel HIV-1 knockdown targets identified by an enriched kinases/phosphatases shRNA library using a long-term iterative screen in Jurkat T-cells. PLoS One 5:e9276. doi:10.1371/journal.pone.0009276 20174665 PMC2822867

[38] Kook I, Jones C. 2016. The serum and glucocorticoid-regulated protein kinases (SGK) stimulate bovine herpesvirus 1 and herpes simplex virus 1 productive infection. Virus Res 222:106–112. doi:10.1016/j.virusres.2016.06.007 27297663

[39] Khorkova O, Stahl J, Joji A, Volmar C-H, Wahlestedt C. 2023. Amplifying gene expression with RNA-targeted therapeutics. Nat Rev Drug Discov 22:539–561. doi:10.1038/s41573-023-00704-7 37253858 PMC10227815

[40] Kwofie KD, Hernandez EP, Kawada H, Koike Y, Sasaki S, Inoue T, Jimbo K, Mikami F, Ladzekpo D, Umemiya-Shirafuji R, Yamaji K, Tanaka T, Matsubayashi M, Alim MA, Dadzie SK, Iwanaga S, Tsuji N, Hatta T. 2023. RNA activation in ticks. Sci Rep 13:9341. doi:10.1038/s41598-023-36523-4 37291173 PMC10250327

[41] Tan CP, Sinigaglia L, Gomez V, Nicholls J, Habib NA. 2021. RNA activation-a novel approach to therapeutically upregulate gene transcription. Molecules 26:6530. doi:10.3390/molecules26216530 34770939 PMC8586927

[42] Beebe NW, Pagendam D, Trewin BJ, Boomer A, Bradford M, Ford A, Liddington C, Bondarenco A, De Barro PJ, Gilchrist J, Paton C, Staunton KM, Johnson B, Maynard AJ, Devine GJ, Hugo LE, Rasic G, Cook H, Massaro P, Snoad N, Crawford JE, White BJ, Xi Z, Ritchie SA. 2021. Releasing incompatible males drives strong suppression across populations of wild and Wolbachia-carrying Aedes aegypti in Australia. Proc Natl Acad Sci U S A 118:e2106828118. doi:10.1073/pnas.2106828118 34607949 PMC8521666

[43] Hussain M, Frentiu FD, Moreira LA, O’Neill SL, Asgari S. 2011. Wolbachia uses host microRNAs to manipulate host gene expression and facilitate colonization of the dengue vector Aedes aegypti. Proc Natl Acad Sci U S A 108:9250–9255. doi:10.1073/pnas.1105469108 21576469 PMC3107320

[44] Parry R, Bishop C, De Hayr L, Asgari S. 2019. Density-dependent enhanced replication of a densovirus in Wolbachia-infected Aedes cells is associated with production of piRNAs and higher virus-derived siRNAs. Virology 528:89–100. doi:10.1016/j.virol.2018.12.006 30583288

[45] Hoffmann AA, Montgomery BL, Popovici J, Iturbe-Ormaetxe I, Johnson PH, Muzzi F, Greenfield M, Durkan M, Leong YS, Dong Y, Cook H, Axford J, Callahan AG, Kenny N, Omodei C, McGraw EA, Ryan PA, Ritchie SA, Turelli M, O’Neill SL. 2011. Successful establishment of Wolbachia in Aedes populations to suppress dengue transmission. Nature New Biol 476:454–457. doi:10.1038/nature10356 21866160

[46] Wiśniewski JR, Zougman A, Nagaraj N, Mann M. 2009. Universal sample preparation method for proteome analysis. Nat Methods 6:359–362. doi:10.1038/nmeth.1322 19377485

[47] Ridley AW, Hereward JP, Daglish GJ, Raghu S, McCulloch GA, Walter GH. 2016. Flight of Rhyzopertha dominica (Coleoptera: Bostrichidae)-a Spatio-Temporal analysis with pheromone trapping and population genetics. J Econ Entomol 109:2561–2571. doi:10.1093/jee/tow226 27986943

[48] Watterson D, Robinson J, Chappell KJ, Butler MS, Edwards DJ, Fry SR, Bermingham IM, Cooper MA, Young PR. 2016. A generic screening platform for inhibitors of virus induced cell fusion using cellular electrical impedance. Sci Rep 6:22791. doi:10.1038/srep22791 26976324 PMC4792136

[49] Pfaffl MW. 2001. A new mathematical model for relative quantification in real-time RT-PCR. Nucleic Acids Res 29:e45. doi:10.1093/nar/29.9.e45 11328886 PMC55695

